# Application of Polyamines and Amino Acid Derivatives Based on 2-Azabicycloalkane Backbone in Enantioselective Aldol Reaction

**DOI:** 10.3390/molecules26175166

**Published:** 2021-08-26

**Authors:** Dominika Iwan, Karolina Kamińska, Elżbieta Wojaczyńska

**Affiliations:** Faculty of Chemistry, Wrocław University of Science and Technology, Wybrzeże Wyspiańskiego 27, 50370 Wrocław, Poland; dominika.iwan@pwr.edu.pl (D.I.); karolina.kaminska@pwr.edu.pl (K.K.)

**Keywords:** aldol reaction, 2-azabicycloalkane, 2-azanorbornane, heterocycle, stereoselectivity

## Abstract

Carbon–carbon bond forming reactions, such as aldol reaction and condensation, belong to extremely desired transformations as manifested by >25,000 entries in SciFinder. Their stereoselective variant requires the use of an appropriate catalyst with a strictly defined structure. Hence, chiral 2-azabicycloalkane-based catalysts were designed, synthesized and tested in a stereoselective aldol reaction between cyclic/acyclic ketone and *p*-nitrobenzaldehyde both in organic and aqueous media. Among catalysts containing a chiral bicyclic backbone, amide based on 2-azabicyclo[3.2.1]octane and pyrrolidine units showed the best catalytic activity and afforded aldol product in excellent chemical yields (up to 95%) and good diastereo- and enantioselectivity (*dr* 22:78, *ee* up to 63%).

Dedicated to Professor Janusz Jurczak in occasion of his 80th birthday.

## 1. Introduction

Dating back to the 19th century, the aldol reaction and its many variants remain an important tool used in organic synthesis for carbon–carbon bond formation and building up molecular complexity and diversity [1,2]. The attractive possibility of creation of two stereocenters in a single step led to the development of stereoselective versions of this transformation [3,4]. To this end, numerous efficient catalysts have been introduced, including chiral metal complexes and organocatalysts. Among the latter, proline and its derivatives are particularly worth mentioning. An initial discovery of usefulness of l-proline in asymmetric aldol-type cyclization was independently reported in the early 1970s by two research groups (>90% enantiomeric excess was noted) [5,6,7]. Since then, this amino acid has been recognized as a versatile organocatalyst in a wide range of asymmetric transformations [8]. In addition, its derivatives, especially prolinamide, were found very useful; the possible mechanism of their action was studied in detail [9,10,11,12]. Protocols engaging various amines were also introduced [13]. In spite of the continuous progress in the field, the existing systems have their drawbacks (e.g., limited substrate scope, access to a particular stereoisomer of the product), and further research is necessary.

**Figure 1:**
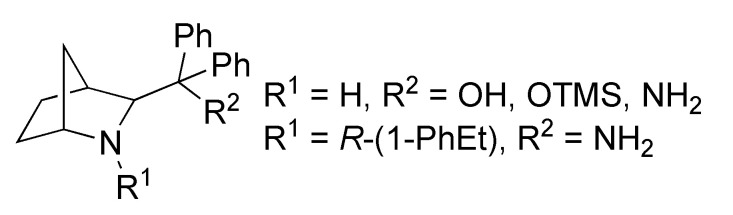
Structure of organocatalysts based on 2-azanorbornane prepared by Nakano’s group.

2-Azanorbornane (2-azabicyclo [2.2.1]heptane) and other 2-azabicycloalkanes with a chiral bicyclic backbone can be used as a versatile platform for the preparation of compounds exhibiting interesting biological activity, and a variety of heterocyclic compounds that have already found application in asymmetric synthesis as ligands or organocatalysts [14]. As an example, a series of derivatives of 2-azanorbornane prepared by Nakano and co-workers (Figure 1) were applied in a stereoselective reaction of isatins with ketones [15]. A catalyst bearing a hydroxyl group led to aldol products in high yields (up to 95%) and modest stereoselectivity (*syn:anti* up to 36:64, up to 64% *ee*).

**Figure 2:**
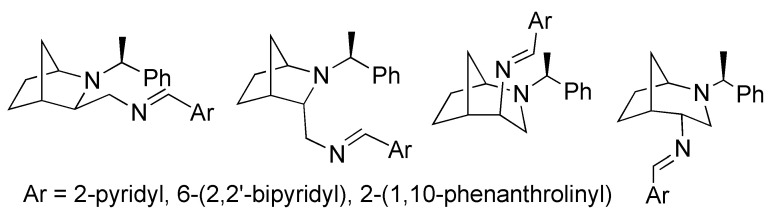
Structure of modular chiral catalysts based on 2-azabicycloalkanes.

In our laboratory, we used 2-azabicyclo[2.2.1]heptane and 2-azabicyclo[3.2.1]octane for the construction of chiral modular catalysts [16]. They were converted to the corresponding, isomeric amines which were reacted with aldehydes bearing pyridine, 2,2′-bipiridine or 1,10-phenanthroline unit. The resulting assemblies (Figure 2) were used in zinc-catalyzed aldol reaction between cyclohexanone and 4-nitrobenzaldehyde. With 10% of Zn(II) complex (formed in situ), good and high yields (up to >95%) were noted, accompanied with *syn/anti* ratio up to 80:20, and *ee* up to 40%. With 4-chlorobenzaldehyde, better stereoselectivity was achieved (*syn*/*anti* > 98:2, *ee*(*syn*) 99%), albeit with much lower yield. Interestingly, in the absence of zinc a modest stereoselectivity was observed as well.

In this communication, we report the application of chiral polyamines, amides and amino acid (proline analogues) based on a 2-azabicycloalkane scaffold as organocatalysts in aldol reaction. We believe that the introduction of this rigid unit bearing multiple stereogenic centers could have a beneficial effect on the stereoselectivity of the process.

## 2. Results and Discussion

**Scheme 1:**
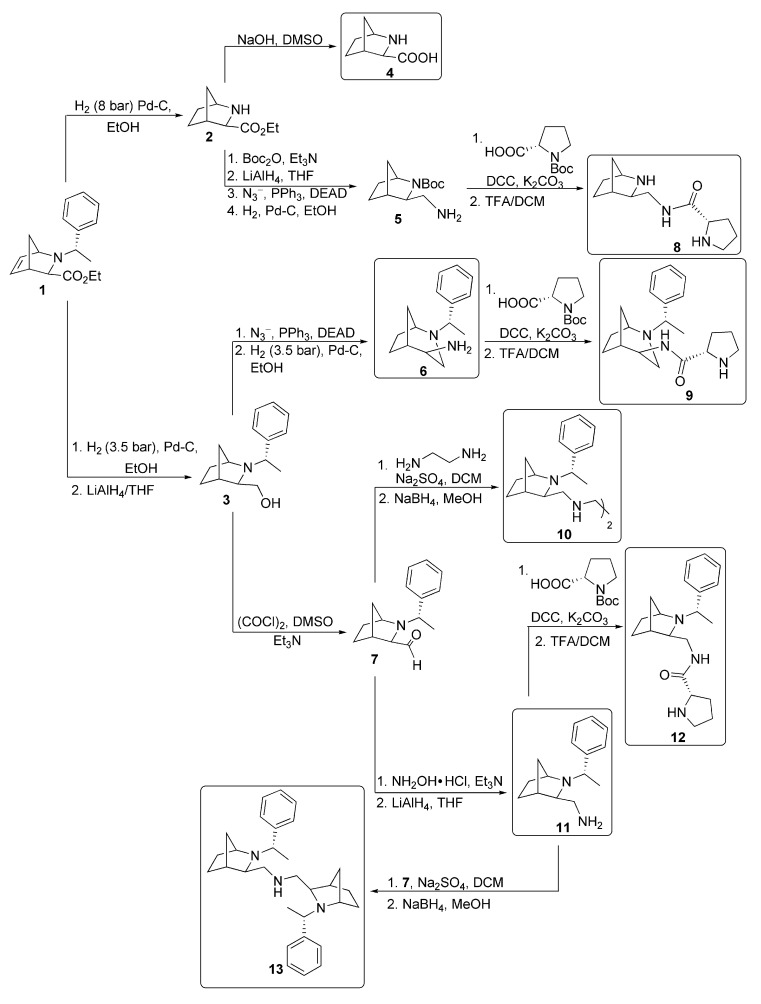
Synthesis of chiral organocatalysts **4**, **6** and **8**–**13**.

In our study we prepared two types of optically active 2-azabicycloalkane organocatalysts, namely polyamines and amides with additional chiral pyrrolidine moiety. Compounds based on a 2-azanorbornane system were obtained by appropriate modifications of the enantiomerically pure aza-Diels-Alder cycloadduct **1** (Scheme 1). This precursor of all studied derivatives, an ester with 2-azanorbornene backbone, was obtained as the major product of the stereoselective *aza*-Diels Alder reaction between cyclopentadiene and a chiral imine [17,18,19,20,21]. Subsequently, compound **1** was subjected to both catalytic hydrogenolysis and catalytic hydrogenation yielding amino ester **2** and hydrogenated product, respectively; the latter was directly transformed into the alcohol **3**. Simple hydrolysis of amino ester **2** led to an amino acid **4** with 2-azanorbornane core, which can be regarded as a bridged proline analogue [14]. The amine group was protected with Boc_2_O in the presence of base followed by the ester group reduction to the alcohol using LiAlH_4_. Next, stereoselective Mitsunobu reaction was performed to transform alcohol into the azide which was readily converted to bisamine **5** with Boc-protected secondary amine group in a bicyclic system [22]. Amine **6** with an expanded bicyclic system of 2-azabicyclo[3.2.1]octane and its 2-azanorbornane-based isomer **11** were prepared from the bicyclic alcohol **3** as described in our previous publications [22,23,24]. Thus, a reaction of **3** with azide under Mitsunobu conditions led to ring expansion [20], yielding—after reduction—a bridged azepane bearing amine function **6**. Swern oxidation followed by transformation of the formed aldehyde **7** to oxime and its reaction with LiAlH_4_ resulted in 2-azabicyclo[2.2.1]heptane derivative bearing amine function **11** [24]. Its reaction with aldehyde **7** and a subsequent reduction with sodium borohydride afforded a *C*_2_-symmetrical triamine **13** in 50% yield, whereas the use of ethylenediamine in the reaction with aldehyde **7** resulted in diimine containing two 2-azanorbornane fragments, which was subsequently reduced to give tetraamine **10** in 70% yield [24].

With bicyclic amines **5**, **6** and **11** in hand, the synthesis of amide-type organocatalysts was feasible. We decided to include in their structure a motif of (*S*)-prolinamide as an additional chiral unit. We intended to investigate the effect of its presence in the catalyst structure on the asymmetric induction in the catalytic aldol reaction. In addition, 2-azabicycloalkane derivatives are considered as bicyclic analogues of l-proline, characterized by greater steric hindrance and the presence of additional stereogenic centers [14]. To obtain amide type organocatalyst, amines **5**, **6** and **11** were reacted with *N*-Boc-l-proline in the presence of DCC coupling reagent and K_2_CO_3_ [24]. The newly prepared amides were fully deprotected using TFA-DCM (1:4 *v/v*) mixture yielding optically pure organocatalysts **8**, **9** and **12** in 64%, 60% and 59% yield, respectively. All of novel compounds have been fully characterized using spectroscopic methods (see Section 3.2.3 and Supplementary Materials).

**Scheme 2:**
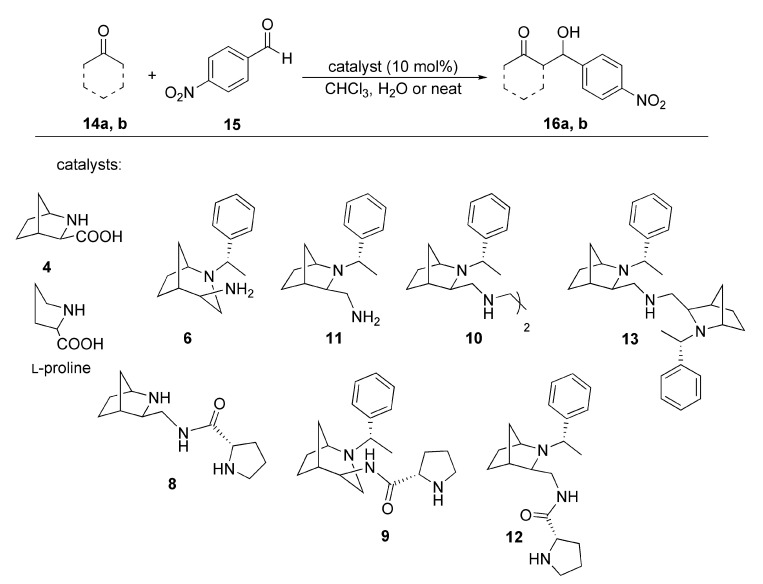
Asymmetric aldol reaction between ketone and *p*-nitrobenzaldehyde using catalysts **4**, **6**, **8**–**13** and l-proline.

We started our catalytic studies from the model aldol reaction between acetone (**14a**) and *p*-nitrobenzaldehyde (**15**) using 10 mol% of the obtained amide type organocatalysts **8**, **9**, **12** at room temperature for 24 h (Scheme 2). We decided to compare their catalytic outcome with well-known catalyst l-proline [5,6,7] and its close bicyclic analogue, α-amino acid **4** (Table 1, entries 1–5). The highest asymmetric induction was observed for proline, and for the bridged azepane-based amide **9** (66% and 60% *ee*, respectively; Table 1, entries 1 and 4). Both catalysts lead for high conversion (for amide **9** prolonging reaction time to 48 h resulted in 90% yield), and favour formation of *R*-enantiomer of the product. In addition, aldol reaction catalyzed with derivative **4** was also very efficient (90% yield), but the stereoselectivity was low (*ee* 22%, Table 1, entry 2). In this case, decreasing the temperature did not improve the enantioselectivity, but only reduced the chemical yield (30% yield at −20 °C). These results indicate that in the presence of additional chiral, bicyclic units in the structure of the organocatalyst the stereodifferentiating properties of l-proline in the aldol reaction are preserved, though not enhanced. A ring-expanded bridged azepane system in combination with pyrrolidine fragment turns out to be favoured over 2-azanorbornane in the structure of organocatalyst, considering the relative efficiency and enantioselectivity (see Table 1, entries 4 and 5). Moreover, the lack of an electron donating substituent at the amine group in the bicyclic unit of amide **8**, responsible also for a large steric hindrance, led to a significant decrease of yield to only 8% (the stereoselectivity was not determined in this case).

Next, we examined aldol reaction with *p*-nitrobenzaldehyde and a cyclic ketone—cyclohexanone, both in organic and aqueous media. For this experiment, we chose amine- and amide-type catalysts **4**, **6**, **8**–**13** possessing bicyclic 2-azacycloalkane frameworks. l-Proline served as a reference in this series as well. To perform the aldol reaction in organic media, we used chloroform as solvent at room temperature. As can be seen from Table 1 (entries 6–14), not all catalysts afforded the expected aldol products. In this experiment, l-proline was inefficient since rather low chemical yield (26%) was accompanied with reasonable stereoselectivity (*syn/anti* = 27:73; entry 6). The diastereomeric ratio was comparable in the case of bicyclic amino acid **4** (*syn/anti* = 21:79), although the yield was even worse (13%; Table 1, entry 7). Similarly, amine-type catalysts with two 2-azanorbornane units **10** and **13** were ineffective in aldol transformation—only traces of products were observed (Table 1, entries 11 and 14). Surprisingly, amines with one bicyclic unit, 2-azabicyclo[3.2.1]octane (**6**) or 2-azabicyclo[2.2.1]heptane (**11**) yielded aldol product with a reversed diastereoselectivity and still not satisfactory yield of 16/43% (Table 1, entries 8 and 13). This time it was 2-azanorbornane that exhibited higher chemical efficiency. Almost complete conversion and good selectivity was observed for amide catalyst **9** (>95% yield, *syn/anti* = 22:78, 57% *ee* for *anti* isomer, entry 10). Attempts of optimization of the reaction conditions by lowering the temperature did not improve the stereoselectivity, but only led to a decrease in the yield of the catalyzed reaction. The amide based on 2-azanorbornane **12** yielded moderate conversion and good diastereoselectivity (63% yield, *syn/anti* = 16:84, ca. 38% *ee*; entry 13). In this case the yield could be improved using lower temperature in aldol reaction (23% yield was noted at room temperature). Again, in the case of an amide **8**, only trace amounts of products were observed (Table 1, entry 9).

Nowadays, the use of water as an environmentally friendly reaction medium is highly recommended. Since proline is not effective in aqueous media, prolinamide derivatives were developed as highly reactive and stereoselective catalysts in either water or brine with a notably low catalyst load [25,26,27,28,29]. In our experiments, the aldol reaction was performed in brine at room temperature, in the presence of acetic acid (20 mol%) as a co-catalyst. The results reveal that only three organocatalysts, **6**, **9** and **11** afforded corresponding aldol product as a diastereomeric mixture. Amine and amide derivatives based on 2-azabicyclo[3.2.1]octane framework **6** and **9** yielded aldol product with almost full conversion (>86%) and good stereoselectivity (**6** *syn/anti* = 35:65, **9** *syn/anti* 27:73, 63% *ee*, entries 17 and 19). Moreover, 2-azanorbornane amine **11** showed notable diastereoselectivity and chemical efficiency (>95% yield, *syn/anti* = 19:81, entry 21). Interestingly, amide with 2-azanorbornane system **12**, a close analogue of **9**, did not promote the aldol reaction—only traces of product were observed (entry 22). Interestingly, the change of the reaction medium resulted in the reversal of diastereoselectivity for the aldol reaction catalysed by compounds **6** and **11** (Table 1, entries 8 vs. 17 and 12 vs. 21), but not for catalyst **9** (Table 1, entries 10 and 19).

Among all of the tested catalysts containing a chiral bicyclic backbone, amide based on 2-azabicyclo[3.2.1]octane **9** provided the corresponding aldol products in excellent chemical yields (up to 95%) and with good stereoselectivity (up to 63% *ee*, *dr* = 22:78, entries 10 and 19) both in organic and aqueous media. These results indicate that introduction of the bicyclic system, both 2-azanorbornane and bridged azepane, into the catalyst structure might be necessary for satisfactory results. Furthermore, the diastereoselection in the reaction relies strongly on the steric interaction. Bicyclic ring system in our catalysts introduces a greater steric hindrance and is more rigid as compared to pyrrolidine moiety of proline. Further research, including catalyst design and structural modifications to improve the stereoselectivity, and mechanistic investigations are in progress.

Since the observed stereochemical outcomes are not outstanding, we checked the possibility of further enantiomeric enrichment of the final products (though at the expense of their amount) by fractioning the samples during their chromatographic purification on a silica column. The effect of self-disproportionation of enantiomers (SDE), well-documented and discussed also in recent *Molecules* papers [30,31,32,33,34], if neglected, can lead to the false determination of stereoselectivity of the asymmetric reaction. On the other hand, it can be beneficial if the optical purity of the original product(s) of an asymmetric reaction is not satisfactory, which might be of particular importance for the compounds exhibiting an interesting biological activity.

First, a sample of aldol product between acetone and *p*-nitrobenzaldehyde was subjected to column chromatography on a silica column, and the sample of original *ee* of 22% was divided into three fractions using *n*-hexane/ethyl acetate 3:1 (*v*/*v*) as the eluent. Their enantiomeric composition was then checked by HPLC analysis (Daicel Chiralpac AS-H column), and the values were in the 20–24% range which suggests a negligible SDE effect in this case. We then decided to examine the products of the aldol reaction between *p*-nitrobenzaldehyde and cyclohexanone. We chose three samples, purified from unreacted substrates and the catalyst, differing in their diastereomeric composition and optical purity of *anti* and *syn* isomers. Each sample was divided into several fractions by passing it through a silica column, and elution with *n*-hexane/ethyl acetate 3:1 (*v*/*v*). The results are collected in Table 2. This time, the samples contained mixtures of diastereomers which were not separated under the conditions used, though we observed that later fractions were significantly enriched in the major one, either *anti* (samples 1 and 3) or *syn* (sample 2). In the case of sample 1, the variation of enantiomeric excess across the fractions of is small (Δ*ee* = 5%), but all values are rather low. Much more pronounced changes in enantiomeric composition were observed, however, for samples 2 and 3: for *anti* form Δ*ee* = 18% and 13%, respectively, and for *syn* isomer even 42% and 50%! For the latter, the tendency of enantiomeric purity to increase in the late fractions is clearly visible. The observed effects could be attributed to interactions between two diastereomers, but particularly for fractions 3 and 4 of sample 2, containing practically pure *syn* diastereomer and differing much in its *ee*, the occurrence of SDE is the most possible explanation. In conclusion, our observation shows the possibility to enhance the moderate stereochemical outcome of the asymmetric aldol reaction by fractioning the received samples on a silica column, and the importance of determining the global *ee* when the effect of asymmetric reaction is measured. It should be underlined that all data provided in Table 1 refer to non-fractionated samples.

## 3. Materials and Methods

### 3.1. Measurements

All reagents and solvents were acquired from Merck KGaA, Darmstadt, Germany, and used without further purification. Silica gel 60 (70–230 mesh) was used for the chromatographic separation, and silica gel 60 precoated plates (visualized with UV light and by staining with iodine) were applied for thin layer chromatography (TLC). The NMR spectra were recorded at 298 K on Jeol 400yh (Jeol Ltd., Tokyo, Japan) and Bruker Avance II 600 instruments (Bruker, Billerica, MA, USA) at 400 and 600 MHz (^1^H), and 100 and 150 MHz (^13^C), respectively. The signals were referenced to residual solvent signals (chloroform-*d*, methanol-*d*_4_). Optical rotations were measured using an Optical Activity Ltd. Model AA-5 automatic polarimeter (Optical Activity, Ltd., Ramsey, UK). Infrared spectra were recorded in the range of 500–4000 cm^−1^ using a Perkin Elmer 2000 FTIR spectrophotometer (PerkinElmer, Waltham, MA, USA). High resolution mass spectra (HRMS) were collected with electrospray ionization using a Waters LCT Premier XE TOF instrument (Waters Corporation, Milford, MA, USA). Melting points were determined on the Schmelzpunkt Bestimmer Apotec apparatus (WEPA Apothekenbedarf GmbH & Co. KG., Hillscheid, Germany) using a standard open capillary.

### 3.2. Preparation of Compounds

#### 3.2.1. Synthesis of Starting Compounds

Synthesis of *aza*-Diels-Alder cycloadduct **1** and its reduction leading to compounds **2** and **3** was accomplished as described in the literature [20,21,22]. (1*S*,3*R*,4*R*)-2-azabicyclo[2.2.1]heptane-3-carboxylic acid **4** was obtained via alkaline hydrolysis amino ester **2** [35].

#### 3.2.2. Preparation of Polyamines (**5**, **6**, **11**, **10**, **13**)

Synthesis of amines, (1*S*,4*S*,5*R*)-2-[(*S*)-1-phenylethyl]-4-amine-2-azabicyclo[3.2.1]octane **6**, and (1*S*,3*R*,4*R*)-2-[(*S*)-1-phenylethyl]-3-aminemethyl-2-azabicyclo[2.2.1]heptane **11** was achieved as described in our previous publications [21,22,23,24]. Preparation of enantiomerically pure polyamine derivatives **10** and **13** with two 2-azanorbornane subunits was reported as well [24]. Amine **5** was synthesized using known procedure [21,36].

#### 3.2.3. General Procedure for Amide Synthesis

All bicyclic amides **8**, **9**, **12** were synthesized using our previously described methodology [23]. Primary amine (1 mmol), *N*-Boc-l-proline (1.11 mmol), potassium carbonate (2.02 mmol), and *N*,*N*′-dicyclohexylcarbodiimide (DCC, 1.0 mmol) were dissolved in acetonitrile and stirred for 24 h at room temperature. The precipitated (DCU, dicyclohexylurea) was separated, and the solvent was removed under reduced pressure. The residue was chromatographed on a silica gel with CH_2_Cl_2_/MeOH 90:10 (*v*/*v*) as eluent. The resulting amide was deprotected by dissolving in a mixture of TFA/CH_2_Cl_2_ (1:4), and stirring for 2 h at room temperature. The reaction mixture was neutralized using concentrated aqueous ammonia and extracted with CH_2_Cl_2_ or ethyl acetate. Combined organic phases were dried over anhydrous Na_2_SO_4_ and concentrated under vacuum. The residue was purified on the silica with CH_2_Cl_2_/MeOH 90:10 (*v*/*v*) as eluent to give desired amides. Amide **8** was additionally crystallized from diethyl ether.

*N-((1S,3R,4R)-2-azabicyclo[2.2.1]heptan-3-ylmethyl)pyrrolidine-2-carboxamide* (**8**).

White crystals, yield 84.5 mg (64%), mp 65 °C, [α]_D_^20^ = −26.3 (c 0.0057, MeOH). ^1^H NMR (400 MHz, methanol-*d*_4_): δ 1.53–1.57 (m, 1H), 1.71–1.94 (m, 6H), 2.02–2.11 (m, 3H), 2.41–2.48 (m, 1H), 2.58 (br s, 1H), 3.34–3.50 (m, 4H), 4.10 (br s, 1H), 4.30–4.34 (m, 1H) ppm. ^13^C NMR (400 MHz, MeOH-d_4_): δ 27.7, 29.1, 30.9, 33.5, 38.0, 42.5, 44.7, 50.0, 62.4, 63.7, 66.9, 173.6 ppm. HRMS (ESI+, m/z) calcd for [C_12_H_21_N_3_O]^+^ ([M + H]^+^) 224.1763; found 224.1758.

*N-((1S,4S,5R)-2-((S)-1-phenylethyl)-2-azabicyclo[3.2.1]octan-4-yl)pyrrolidine-2-carboxamide* (**9**).

Amide **9** was prepared, characterized and described by our group in previous work [23].

*N-(((1S,3R,4R)-2-(1-phenylethyl)-2-azabicyclo[2.2.1]heptan-3-yl)methyl)pyrrolidine-2-carboxamide* (**12**).

Yellow oil, yield 0.39 g (62%). [α]_D_^20^ = 42.6 (c 0.60, CH_2_Cl_2_). ^1^H NMR (400 MHz, CDCl_3_): δ 1.19–1.26 (m, 3H), 1.29–1.41 (m, 4H), 1.54–1.66 (m, 3H), 1.71–1.78 (m, 1H), 1.99–2.08 (m, 3H), 2.38 (t, 2H, J = 5.6 Hz), 2.59 (br s, 1H), 2.81–2.86 (m, 1H), 2.92–2.98 (m, 1H), 3.47 (q, 1H, J = 6.4 Hz), 3.56–3.60 (m, 1H), 3.65 (br s, 1H), 7.19–7.36 (m, 5H) ppm. ^13^C NMR (75 MHz, CDCl_3_) δ = 22.3, 22.6, 26.3, 29.4, 30.7, 35.6, 41.1, 42.8, 47.3, 59.0, 60.6, 61.3, 68.9, 127.6, 128.1, 128.4, 145.7, 174.4 ppm. HRMS (ESI+, m/z): calcd for C_20_H_29_N_3_O ([M+H]^+^) 328.2389; found 328.2379.

#### 3.2.4. Catalytic Reactions

**Typical procedure for aldol reaction with acetone (Table 1, entries 1–5).**

A catalyst (0.05 mmol) was dissolved in 5 mL of acetone. The solution was stirred at room temperature for 15 min in closed system, and *p*-nitrobenzaldehyde (75.5 mg, 0.5 mmol) was added. After 24 h the sample of 100 µL was withdrawn and diluted in 1 mL of CH_2_Cl_2_ to measure the *ee* using HPLC. The reaction was finished by evaporation of acetone and chromatographed on the silica with eluent *n*-hexane/ethyl acetate 3:1 (*v*/*v*). The enantiomeric excess of 4-hydroxy-4-(4-nitrophenyl)butan-2-one was determined by HPLC analysis; the results for crude samples and the samples after chromatography were practically identical (within experimental error).

*4-Hydroxy-4-(4-nitrophenyl)butan-2-one.*

^1^H NMR (CDCl_3_, 400 MHz) δ 2.20 (s, 3H), 2.83–2.85 (m, 2H), 3.66 (br s, 1H), 5.24 (t, 1H, *J =* 6.4 Hz), 7.50–7.52 (2H, m), 8.14–8.16 (2H, m) ppm. Enantiomeric excess determined by chiral HPLC analysis. Chiralpak AS-H column, *n*-hexane/isopropanol 85:15 (*v*/*v*), 1.0 mL/min, λ = 254 nm; t_1_ = 25.9 min (*R*-enantiomer), t_2_ = 34.9 min (*S*-enantiomer). The absolute configuration was assigned based on literature data [37].

**Aldol reaction with between *p*-nitrobenzaldehyde and cyclohexanone in CHCl_3_ (Table 1, entries 6–14).**

A mixture of catalyst (0.050 mmol, 0.10 eq.), cyclohexanone (0.26 mL, 2.5 mmol, 5.0 eq,) in 1.0 mL of CHCl_3_ was stirred at room temperature for 15 min in a closed flask, and *p*-nitrobenzaldehyde (76 mg, 0.50 mmol) was added. After 24 h the reaction was finished by evaporation of solvent. Raw mixture was chromatographed on the silica, and eluted with *n*-hexane/ethyl acetate 3:1 (*v*/*v*). The enantiomeric excess of 2-(hydroxy(4-nitrophenyl)methyl)cyclohexanone was determined by HPLC analysis.

*2-(Hydroxy(4-nitrophenyl)methyl)cyclohexanone.*

*Syn* diastereomer: ^1^H NMR (CDCl_3_, 400 MHz) δ 1.50–2.13 (m, 6H), 2.39–2.50 (m, 2H), 2.61–2.65 (m, 1H), 3.20 (s, 1H), 5.48 (m, 1H), 7.49–7.51 (2H), 8.18–8.21 (m, 2H) ppm. Enantiomeric excess determined by chiral HPLC analysis, with Chiralpak AD-H column, *n*-hexane/isopropanol 95:5 (*v*/*v*), 1.0 mL/min, λ = 254 nm; t_1_ = 27.0 min, t_2_ = 32.0 min. *Anti* diastereomer: ^1^H NMR (CDCl_3_, 400 MHz) δ 1.29–2.09 (m, 6H), 2.30–2.47 (m, 2H), 2.55–2.61 (m, 1H), 4.10 (s, 1H), 4.87 (d, *J* = 8.0 Hz, 2H), 7.48 (m, 2H), 8.16 (m, 2H) ppm. Enantiomeric excess determined by chiral HPLC analysis, with Chiralpak AD-H column, n-hexane/isopropanol 95:5 (*v*/*v*), 1.0 mL/min, λ = 254 nm; t_1_ = 36.4 min, t_2_ = 49.4 min.

**Aldol reaction with between *p*-nitrobenzaldehyde and cyclohexanone in brine (Table 1, entries 15–23).**

A mixture of catalyst (0.050 mmol, 0.10 eq.), cyclohexanone (0.26 mL, 2.5 mmol, 5.0 eq,) and acetic acid (5.8 µL, 0.10 mmol, 0.20 eq.) in 1.0 mL of brine was stirred at room temperature for 15 min in a closed flask. *p*-Nitrobenzaldehyde (76 mg, 0.50 mmol) was then added. After 24 h the reaction was quenched with saturated ammonium chloride solution and extracted three times with ethyl acetate. Combined organic phases were dried over anhydrous Na_2_SO_4_ and concentrated under vacuum. The residue was purified on the silica column with *n*-hexane/ethyl acetate 3:1 (*v*/*v*) as the eluent. The enantiomeric excess of 2-(hydroxy(4-nitrophenyl)methyl)cyclohexanone was determined by HPLC analysis.

## 4. Conclusions

We have demonstrated the utility of the chiral organocatalysts equipped in primary, secondary or/and tertiary amine, and amide functionality in the asymmetric aldol reaction. A structure-based screening of catalytic activity of these bicyclic compounds revealed that *N*-substitution of 2-azabicycloalkane is necessary for their efficiency. Polyamines were found inefficient, but monomeric amines and corresponding amides showed the outcome comparable with l-proline. An interesting possibility of reversal of *syn*/*anti* diastereoselectivity by using either the isomer with six- or seven-membered ring, or by the change of reaction medium (chloroform vs. brine) is worth underlining. Furthermore, these 2-azabicycloalkane-based catalysts can be still functionalized, and are available in various stereoisomeric forms (epimers, enantiomers). Taking into account the capacity of the described chiral heterocycles to act as catalysts, their potential in asymmetric synthesis remains to be fully examined.

## Figures and Tables

**Table 1:** Results of aldol reaction of **14a, b** and **15** using organocatalysts **4**, **6**, **8–13** and l-proline.

Entry	Catalyst	Ketone	Solvent	Yield [%] ^d^	*anti*/*syn*^e^	*ee* [%] ^f^
1 ^a^	l-proline	acetone	neat	>95	-	66 (R)
2 ^a^	**4**	acetone	neat	89	-	22 (R)
3 ^a^	**8**	acetone	neat	<8	-	-
4 ^a^	**9**	acetone	neat	59 g	-	60 (R)
5 ^a^	**12**	acetone	neat	15	-	52 (R)
6 ^b^	l-proline	cyclohexanone	CHCl3	26	73:27	*(anti)* 63*(syn)* 56
7 ^b^	**4**	cyclohexanone	CHCl3	13	79:21	*(anti)* 11*(syn)* 6
8 ^b^	**6**	cyclohexanone	CHCl3	16	26:74	*(anti)* 22*(syn)* 0
9 ^b^	**8**	cyclohexanone	CHCl3	traces	-	-
10 ^b^	**9**	cyclohexanone	CHCl3	>95 h	78:22	*(anti)* 57*(syn)* 15
11 ^b^	**10**	cyclohexanone	CHCl3	traces	-	-
12 ^b^	**11**	cyclohexanone	CHCl3	43	26:74	*(anti)* 20*(syn)* 21
13 ^b^	**12**	cyclohexanone	CHCl3	63 e	84:16	*(anti)* 38*(syn)* 40
14 ^b^	**13**	cyclohexanone	CHCl3	0	-	-
15 ^c^	l-proline	cyclohexanone	brine	traces	-	-
16 ^c^	**4**	cyclohexanone	brine	traces	-	-
17 ^c^	**6**	cyclohexanone	brine	86	65:35	*(anti)* 16*(syn)* 31
18 ^c^	**8**	cyclohexanone	brine	traces	-	-
19 ^c^	**9**	cyclohexanone	brine	>95	73:27	*(anti)* 63*(syn)* 44
20 ^c^	**10**	cyclohexanone	brine	traces	-	-
21 ^c^	**11**	cyclohexanone	brine	>95	81:19	*(anti)* 3*(syn)* 14
22 ^c^	**12**	cyclohexanone	brine	traces	-	-
23 ^c^	**13**	cyclohexanone	brine	traces	-	-

^a^ Unless otherwise specified, the reaction was carried out in neat acetone (5 mL), in the presence of 10 mol% organocatalysts, at room temperature for 24 h. (See Section 3.2.4); ^b^ unless otherwise specified, the reaction was carried out in CHCl_3_ (1 mL), in the presence of 10 mol% organocatalysts, at room temperature for 24 h. (See Section 3.2.4); ^c^ unless otherwise specified, the reaction was carried out in brine (1 mL), in the presence of 10 mol% organocatalysts, and 20 mol% HOAc, at room temperature for 24 h. (See Section 3.2.4); ^d^ isolated yields (a mixture of diastereomers); ^e^ Diastereoselectivity was determined by HPLC analysis of the reaction mixture; ^f^ the *ee* values for the reaction with acetone were determined by HPLC analysis with a Daicel Chiralpac AS-H column, and for the reaction with cyclohexanone—with a Daicel Chiralpac AD-H column; ^g^ Reaction was performed at −20 °C; ^h^ reaction was performed at 0 °C.

**Table 2:** Stereochemical effects of chromatography of aldol adducts obtained in the reaction between cyclohexanone and *p*-nitrobenzaldehyde ^a^.

Sample	Fractions	Mass/mg	*dr* (*anti:syn*)	*ee* (*anti*)/%	*ee* (*syn*)/%
1	Raw mixture	16.0	88:12	3	11
	1	1.6	85:15	5	11
	2	5.5	95:5	2	14
	3	1.7	97:3	2	9
	4	5.4	97:3	2	12
	5	1.7	97:3	2	11
2	Raw mixture	60.0	24:76	57	15
	1	8.6	33:67	nd ^b^	
	2	27.5	19:81	61	4
	3	18.6	1:99	43	15
	4	2.9	1:99	52	46
3	Raw mixture	48.0	80:20	54	52
	1	16.9	57:43	nd ^b^	
	2	17.6	88:12	48	6
	3	13.5	98:2	61	56

^a^ Stationary phase: silica gel 60 (70–230 mesh), mobile phase: *n*-hexane/ethyl acetate 3:1 (*v*/*v*). Both *dr* and *ee* determined by HPLC (Chiralpak AD-H column). ^b^ Not determined due to signal overlap in the HPLC trace.

## Data Availability

Not applicable.

## Supplementary Materials

The following are available online. Experimental data and ^1^H and ^13^C-NMR spectra of novel compounds.
