# Supplementary material for: Application of Polyamines and Amino Acid Derivatives Based on 2-Azabicycloalkane Backbone in Enantioselective Aldol Reaction
Source: Molecules. 2021 Aug 26;26(17):5166. doi: 10.3390/molecules26175166 (PMC8434137; doi:10.3390/molecules26175166)
Supplement: Supplementary file 1 [file molecules-26-05166-s001.zip › molecules-1360179-supplementary.pdf]

**Supplementary Material**

# Application of Polyamines and Amino Acid Derivatives Based on 2-Azabicycloalkane Backbone in a Stereoselective Aldol Reaction

**Dominika Iwan, Karolina Kamińska, Elżbieta Wojaczyńska***

Faculty of Chemistry, Wrocław University of Science and Technology, Wybrzeże Wyspiańskiego 27, 50370 Wrocław, Poland

*Correspondence: elzbieta.wojaczynska@pwr.edu.pl; Tel.: +48-713-202-410

# Content

1.General Information....................................................................................................3

2.Copies of HPLC Chromatograms ....................................................................................4

3.NMR Spectra of Catalyst **8** and **12** ..........................................................................12

# 1. General Information

All commercial reagents were purchased and used without further purification. All reactions were carried out in flame-dried glassware with magnetic stirring. Schmelzpunkt Bestimmer Apotec melting point apparatus (WEPA Apothekenbedarf GmbH & Co. KG., Hillscheid, Germany) was used for the determination of melting points in a standard open capillary. $^{1}H$ and $^{13}C$ NMR spectra were recorded on Jeol 400yh (Jeol Ltd., Tokyo, Japan), Bruker Avance III 500, and Bruker Avance II 600 spectrometers (Bruker, Billerica, MA, USA). The residual $^{1}H$ or $^{13}C$ signals of the solvent ($CDCl_3$, MeOH-$d_4$) were used as references. Multiplicity of chemical shift are reported as s = singlet, d = doublet, t = triplet, q = quartet, dd = doublet of doublets, m = multiplet and br = broad. Chemical shifts are given in ppm, and coupling constants are expressed in Hz. The spectra are shown in Supplementary Material. High-resolution mass spectra were recorded using electrospray ionization mode on a Waters LCT Premier XE TOF spectrometer (Waters Corporation, Milford, MA, USA). Infrared spectra were measured in a 4000–400 $cm^{-1}$ range on a Perkin Elmer 2000 FTIR instrument (PerkinElmer, Waltham, MA, USA). Optical rotations were determined with an automatic polarimeter Model AA-5 (OpticalActivity, Ltd., Ramsey, UK); $[\alpha]^{20}_{D}$ values are expressed in $10^{-1}$ deg $cm^2$ $g^{-1}$. Silica gel 60 (60–200 μm, 70–230 mesh) was used for column chromatography, and precoated plates precoated with the same adsorbent were applied for thin-layer chromatography. Diastereomeric ratio and enantiomeric excess were determined by high performance liquid chromatography (HPLC) with chiral columns (Chiralpak ASH and ADH).

## 2. Copies of HPLC Chromatograms

Racemic mixture

Chiralpak AS-H, hexane/isopropanol 85:15, $\lambda$ = 254 nm, 1.0 ml/min

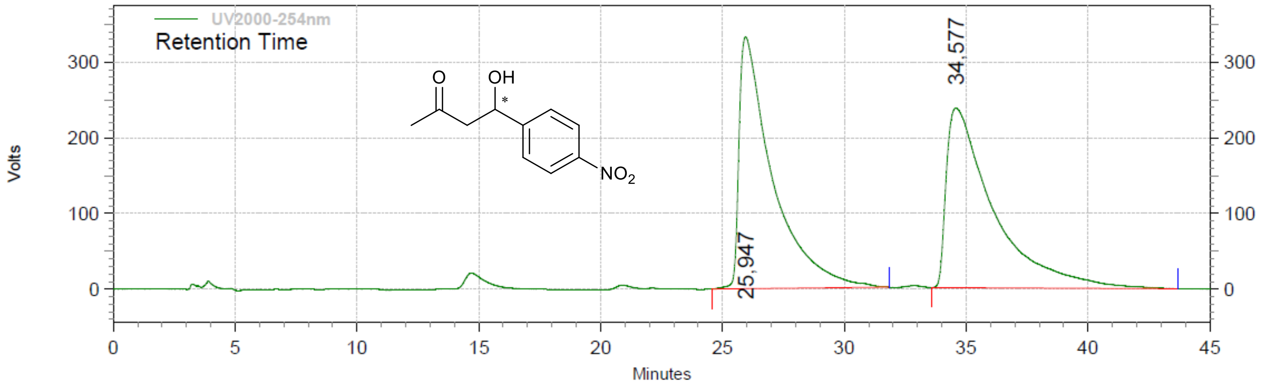


| Retention Time | Area | Area % | Height | Height % |
|---|---|---|---|---|
| 25,947 | 31662683 | 49,83 | 332746 | 58,35 |
| 34,577 | 31884766 | 50,17 | 237467 | 41,65 |

L-Proline, entry 1 in Table 1.

10% mol of L-proline, in acetone at RT

Chiralpak AS-H, hexane/isopropanol 85:15, $\lambda$ = 254 nm, 1.0 ml/min

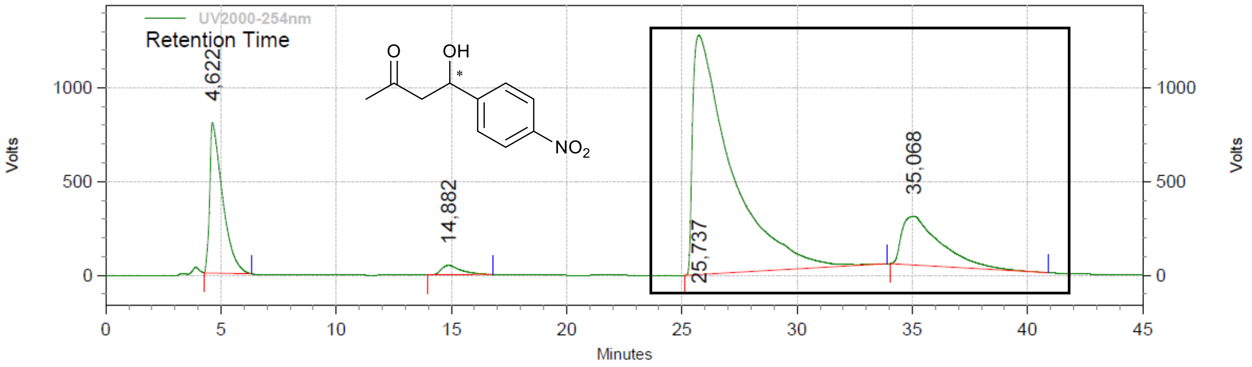


| Retention Time | Area | Area % | Height | Height % |
|---|---|---|---|---|
| 4,622 | 31136529 | 14,69 | 802669 | 33,61 |
| 14,882 | 2868562 | 1,35 | 49269 | 2,06 |
| 25,737 | 147501851 | 69,59 | 1276016 | 53,43 |
| 35,068 | 30446184 | 14,36 | 260415 | 10,90 |

Compound **4**, entry 2 in Table 1.

10% mol of cat **4**, in acetone at RT
Chiralpak AS-H, hexane/isopropanol 85:15, $\lambda$ = 254 nm, 1.0 ml/min

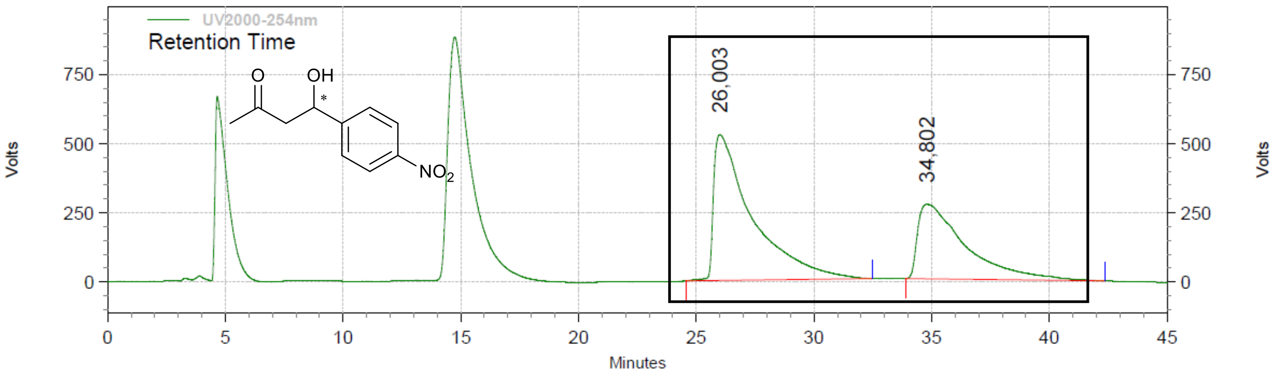


| Retention Time | Area | Area % | Height | Height % |
|---|---|---|---|---|
| 26,003 | 60434036 | 61,24 | 527900 | 66,13 |
| 34,802 | 38251800 | 38,76 | 270366 | 33,87 |

Compound **9**, entry 4 in Table 1.

10% mol of cat **9**, in acetone at RT
Chiralpak AS-H, hexane/isopropanol 85:15, $\lambda$ = 254 nm, 1.0 ml/min

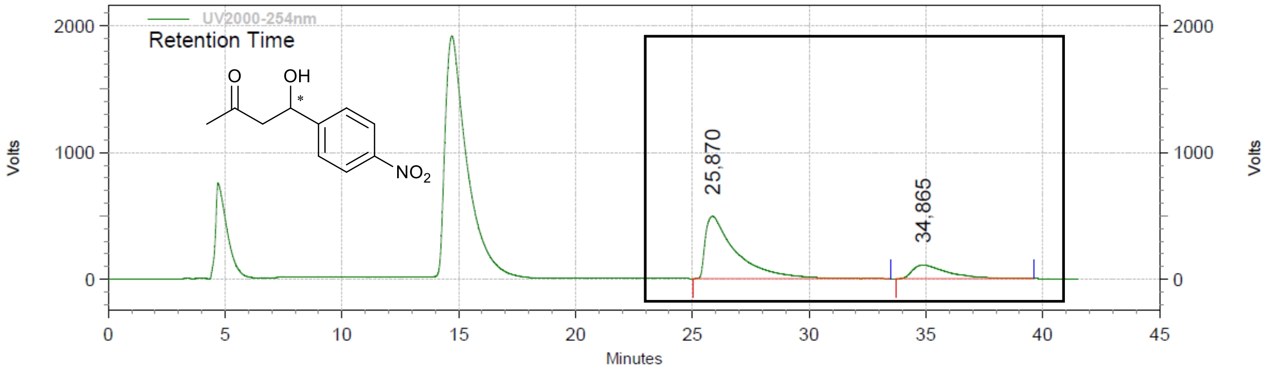


| Retention Time | Area | Area % | Height | Height % |
|---|---|---|---|---|
| 25,870 | 45181056 | 79,86 | 492286 | 82,23 |
| 34,865 | 11393450 | 20,14 | 106380 | 17,77 |

Compound **12**, entry 5 in Table 1.

10% mol of cat **12**, in acetone at RT

Chiralpak AS-H, hexane/isopropanol 85:15, $\lambda$ = 254 nm, 1.0 ml/min

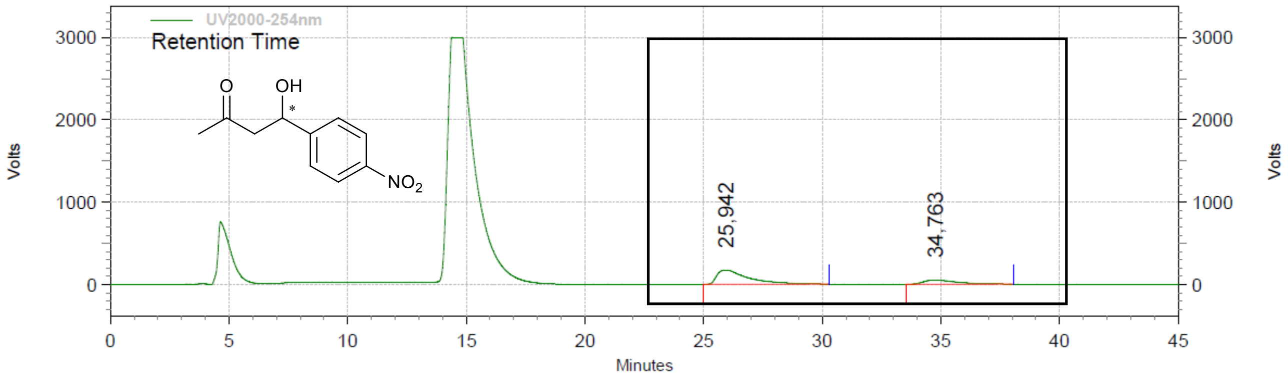


| Retention Time | Area | Area % | Height | Height % |
|---|---|---|---|---|
| 25,942 | 16050395 | 75,91 | 172561 | 76,96 |
| 34,763 | 5094183 | 24,09 | 51662 | 23,04 |

Racemic mixture

Chiralpak AD-H, hexane/isopropanol 95:5, $\lambda$ = 254 nm, 1.0 ml/min

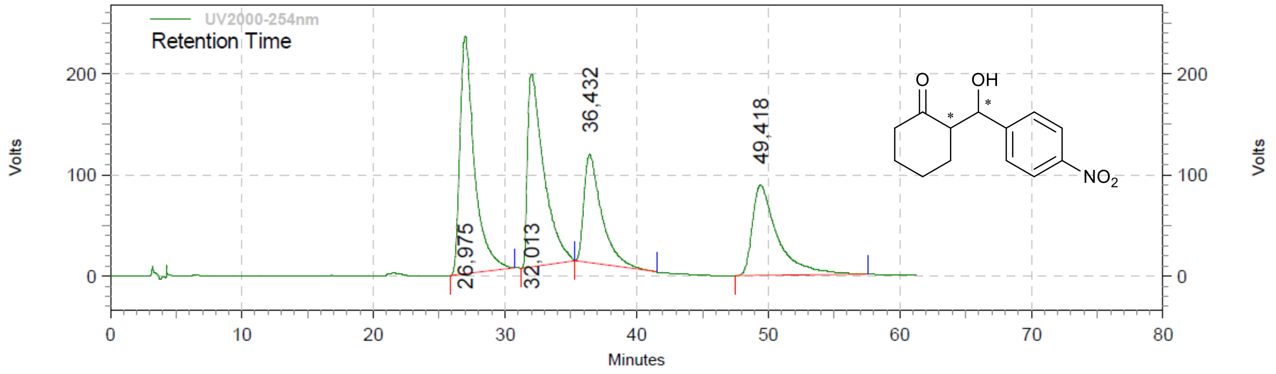


| Retention Time | Area | Area % | Height | Height % |
|---|---|---|---|---|
| 26,975 | 18019742 | 31,96 | 235088 | 37,88 |
| 32,013 | 16480261 | 29,23 | 189980 | 30,61 |
| 36,432 | 9928603 | 17,61 | 106711 | 17,19 |
| 49,418 | 11955573 | 21,20 | 88817 | 14,31 |

Compound **4**, entry 7 in Table 1.

10% mol of cat **4**, in CHCl3 at RT

Chiralpak AD-H, hexane/isopropanol 95:5, $\lambda$ = 254 nm, 1.0 ml/min

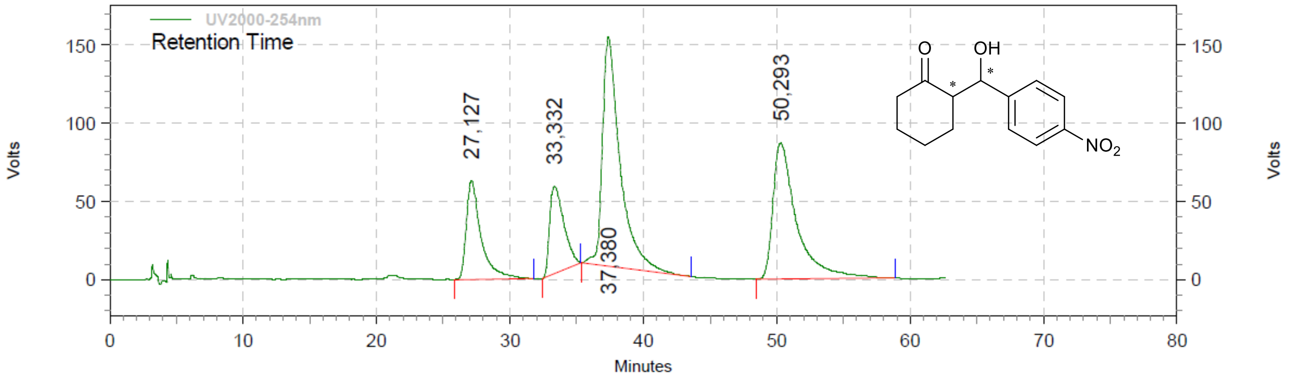


| Retention Time | Area | Area % | Height | Height % |
|---|---|---|---|---|
| 27,127 | 4883342 | 14,66 | 63169 | 17,90 |
| 33,332 | 4148033 | 12,45 | 55886 | 15,83 |
| 37,380 | 13476359 | 40,45 | 146968 | 41,64 |
| 50,293 | 10806146 | 32,44 | 86938 | 24,63 |

Compound **6**, entry 8 in Table 1.

10% mol of cat **6**, in CHCl3 at RT

Chiralpak AD-H, hexane/isopropanol 95:5, $\lambda$ = 254 nm, 1.0 ml/min

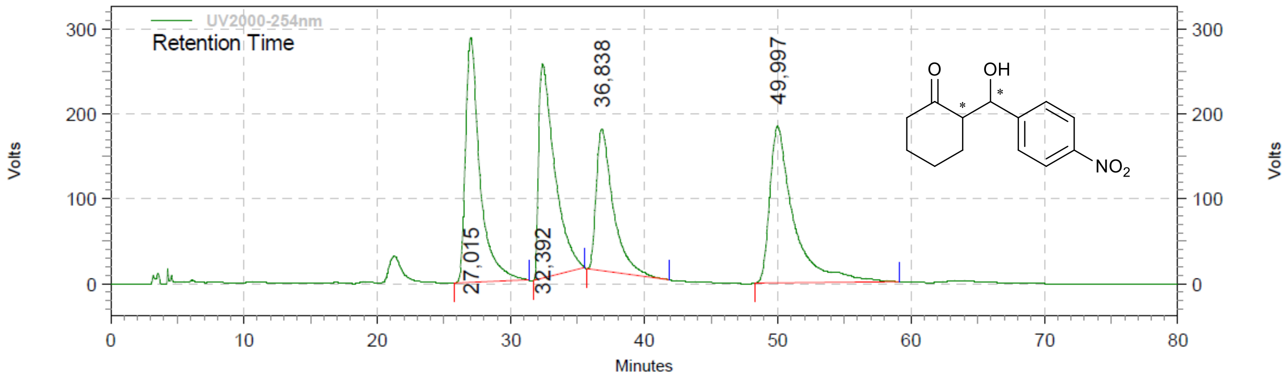


| Retention Time | Area | Area % | Height | Height % |
|---|---|---|---|---|
| 27,015 | 21720497 | 26,87 | 288765 | 32,31 |
| 32,392 | 21168546 | 26,19 | 252484 | 28,25 |
| 36,838 | 14761002 | 18,26 | 167249 | 18,71 |
| 49,997 | 23191765 | 28,69 | 185290 | 20,73 |

Compound **9**, entry 10 in Table 1.

10% mol of cat **9**, in $CHCl_3$ at RT
Chiralpak AD-H, hexane/isopropanol 95:5, $\lambda$ = 254 nm, 1.0 ml/min

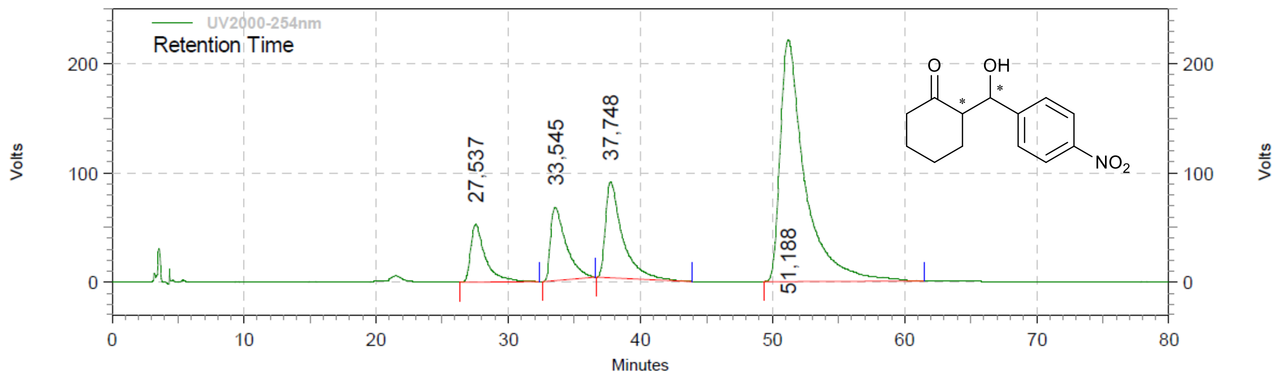


| Retention Time | Area | Area % | Height | Height % |
|---|---|---|---|---|
| 27,537 | 4082268 | 8,82 | 52729 | 12,27 |
| 33,545 | 5547946 | 11,98 | 67205 | 15,64 |
| 37,748 | 7919310 | 17,10 | 87997 | 20,48 |
| 51,188 | 28753002 | 62,10 | 221643 | 51,60 |

Compound **11**, entry 12 in Table 1.

10% mol of cat **11**, in $CHCl_3$ at RT
Chiralpak AD-H, hexane/isopropanol 95:5, $\lambda$ = 254 nm, 1.0 ml/min

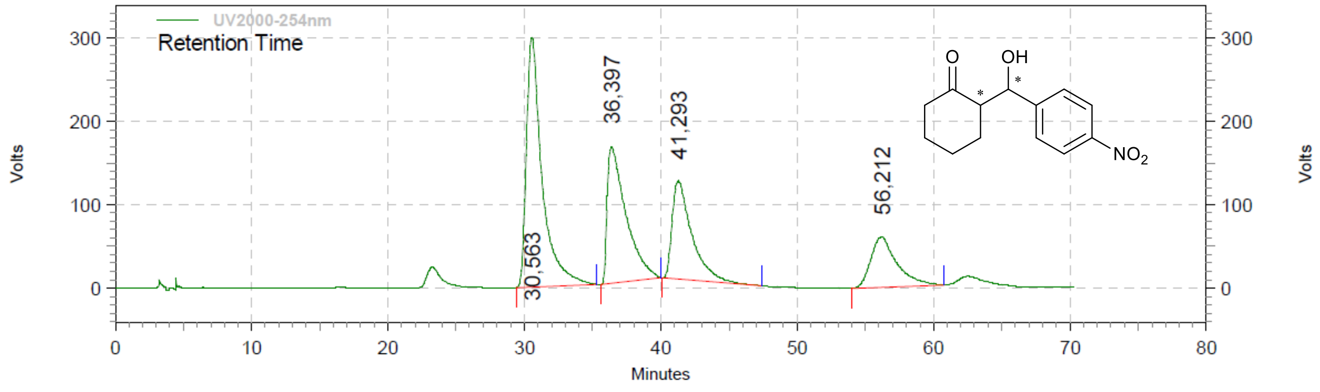


| Retention Time | Area | Area % | Height | Height % |
|---|---|---|---|---|
| 30,563 | 24042476 | 40,31 | 299711 | 46,66 |
| 36,397 | 15693276 | 26,31 | 164310 | 25,58 |
| 41,293 | 11915135 | 19,98 | 118013 | 18,37 |
| 56,212 | 7992036 | 13,40 | 60275 | 9,38 |

Compound **12**, entry 13 in Table 1.

10% mol of cat **12**, in $CHCl_3$ at RT
Chiralpak AD-H, hexane/isopropanol 95:5, $\lambda$ = 254 nm, 1.0 ml/min

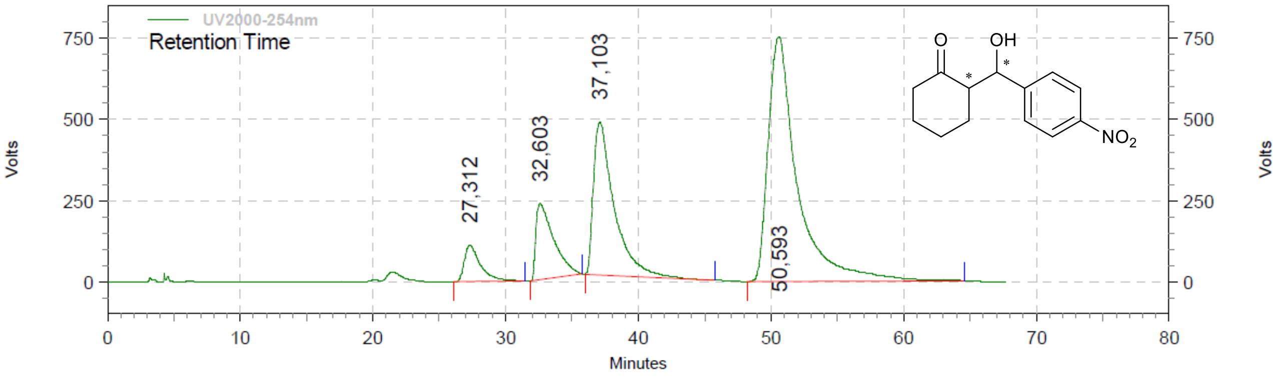


| Retention Time | Area | Area % | Height | Height % |
|---|---|---|---|---|
| 27,312 | 9488431 | 4,81 | 111311 | 7,11 |
| 32,603 | 21918533 | 11,12 | 234077 | 14,94 |
| 37,103 | 51059117 | 25,90 | 469625 | 29,98 |
| 50,593 | 114698791 | 58,17 | 751363 | 47,97 |

Compound **6**, entry 17 in Table 1.

10% mol of cat **6**, 20% mol HOAc, in brine at RT
Chiralpak AD-H, hexane/isopropanol 95:5, $\lambda$ = 254 nm, 1.0 ml/min

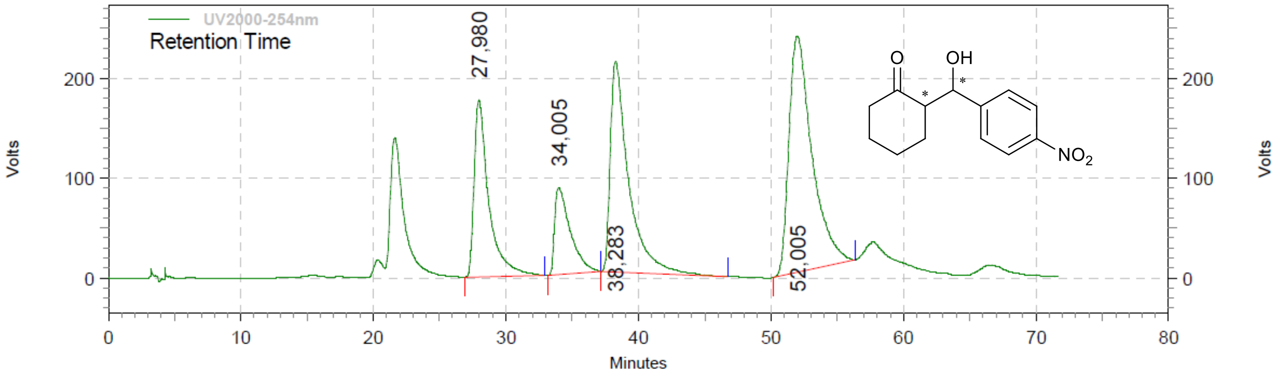


| Retention Time | Area | Area % | Height | Height % |
|---|---|---|---|---|
| 27,980 | 13634118 | 19,54 | 177659 | 24,90 |
| 34,005 | 7180173 | 10,29 | 87492 | 12,26 |
| 38,283 | 20352550 | 29,17 | 211439 | 29,64 |
| 52,005 | 28593847 | 40,99 | 236883 | 33,20 |

Compound **9**, entry 19 in Table 1.

10% mol of cat **9**, 20% mol HOAc, in brine at RT
Chiralpak AD-H, hexane/isopropanol 95:5, $\lambda$ = 254 nm, 1.0 ml/min

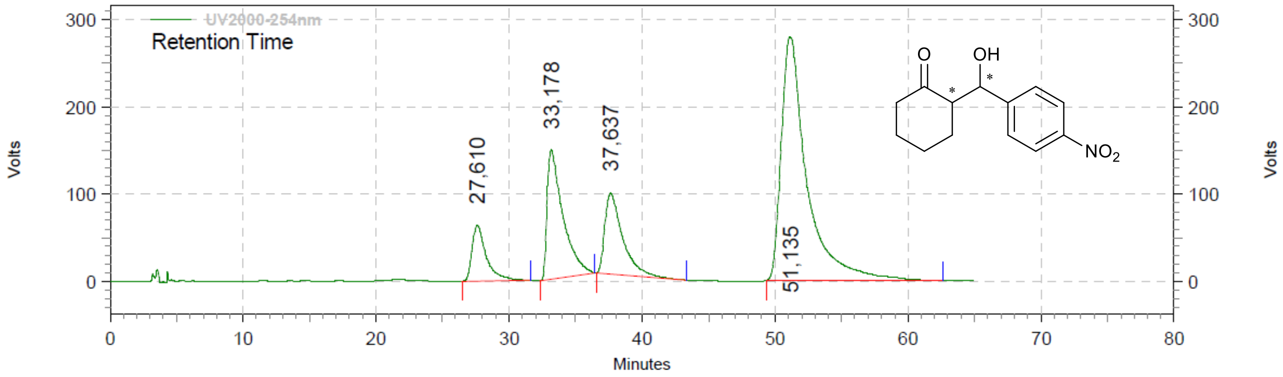


| Retention Time | Area | Area % | Height | Height % |
|---|---|---|---|---|
| 27,610 | 4828495 | 7,60 | 64233 | 10,97 |
| 33,178 | 12317926 | 19,39 | 148056 | 25,29 |
| 37,637 | 8650584 | 13,62 | 92998 | 15,88 |
| 51,135 | 37739298 | 59,40 | 280253 | 47,86 |

Compound **11**, entry 21 in Table 1.

10% mol of cat **11**, 20% mol HOAc, in brine at RT
Chiralpak AD-H, hexane/isopropanol 95:5, $\lambda$ = 254 nm, 1.0 ml/min

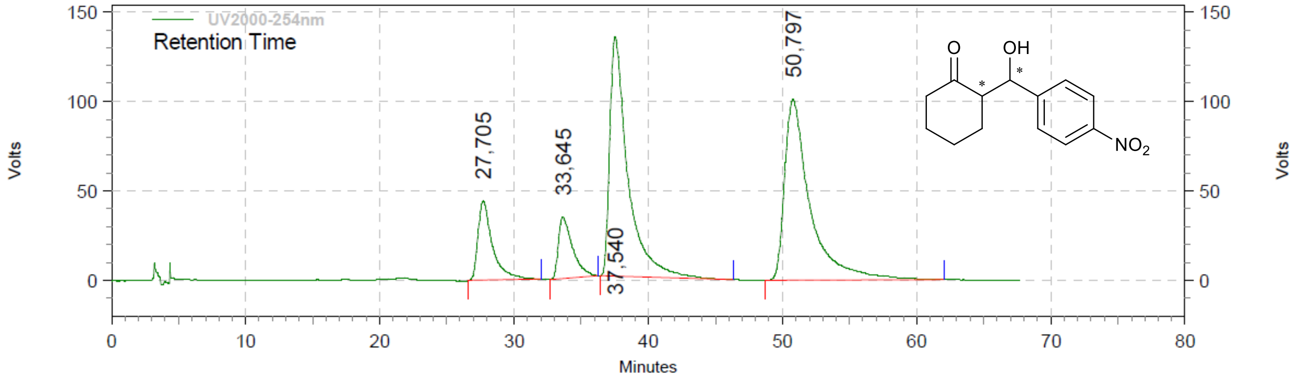


| Retention Time | Area | Area % | Height | Height % |
|---|---|---|---|---|
| 27,705 | 3361346 | 10,51 | 44262 | 14,12 |
| 33,645 | 2525043 | 7,90 | 34119 | 10,88 |
| 37,540 | 12613926 | 39,46 | 133951 | 42,73 |
| 50,797 | 13468111 | 42,13 | 101172 | 32,27 |

Compound **11**, entry 21 in Table 1.

10% mol of cat **11**, 20% mol HOAc, in brine at RT
Chiralpak AD-H, hexane/isopropanol 95:5, λ = 254 nm, 1.0 ml/min

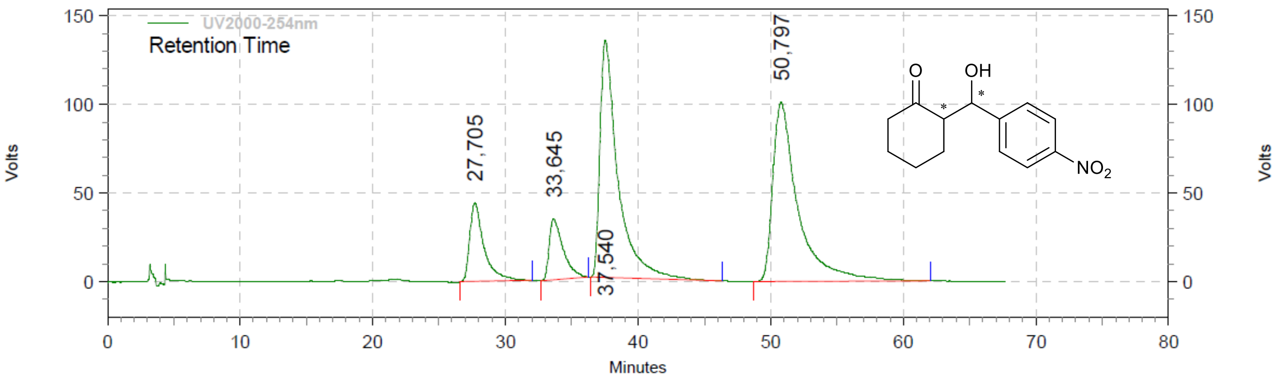


| Retention Time | Area | Area % | Height | Height % |
|---|---|---|---|---|
| 27,705 | 3361346 | 10,51 | 44262 | 14,12 |
| 33,645 | 2525043 | 7,90 | 34119 | 10,88 |
| 37,540 | 12613926 | 39,46 | 133951 | 42,73 |
| 50,797 | 13468111 | 42,13 | 101172 | 32,27 |

## 3. NMR Spectra of Catalysts **8** and **12**

*N-((1S,3R,4R)-2-azabicyclo[2.2.1]heptan-3-ylmethyl)pyrrolidine-2-carboxamide* (**8**)

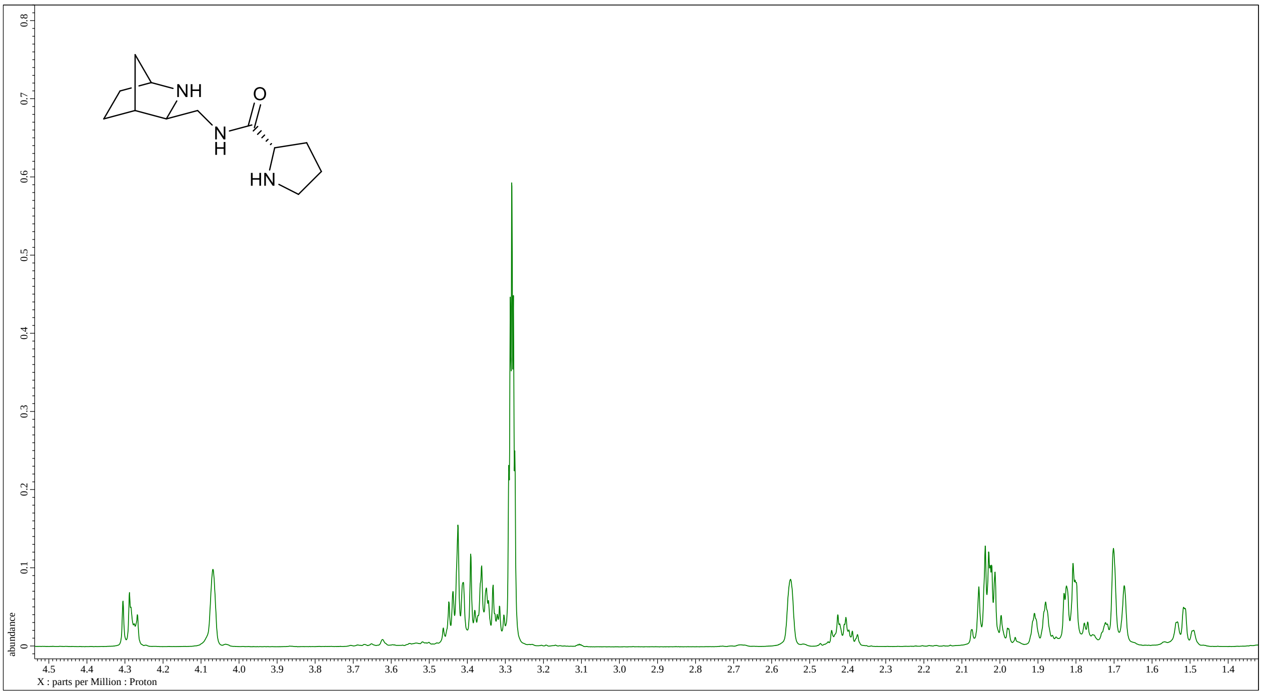


$^{1}$H NMR spectrum of *N*-((1*S*,3*R*,4*R*)-2-azabicyclo[2.2.1]heptan-3-ylmethyl)pyrrolidine-2-carboxamide (**8**)

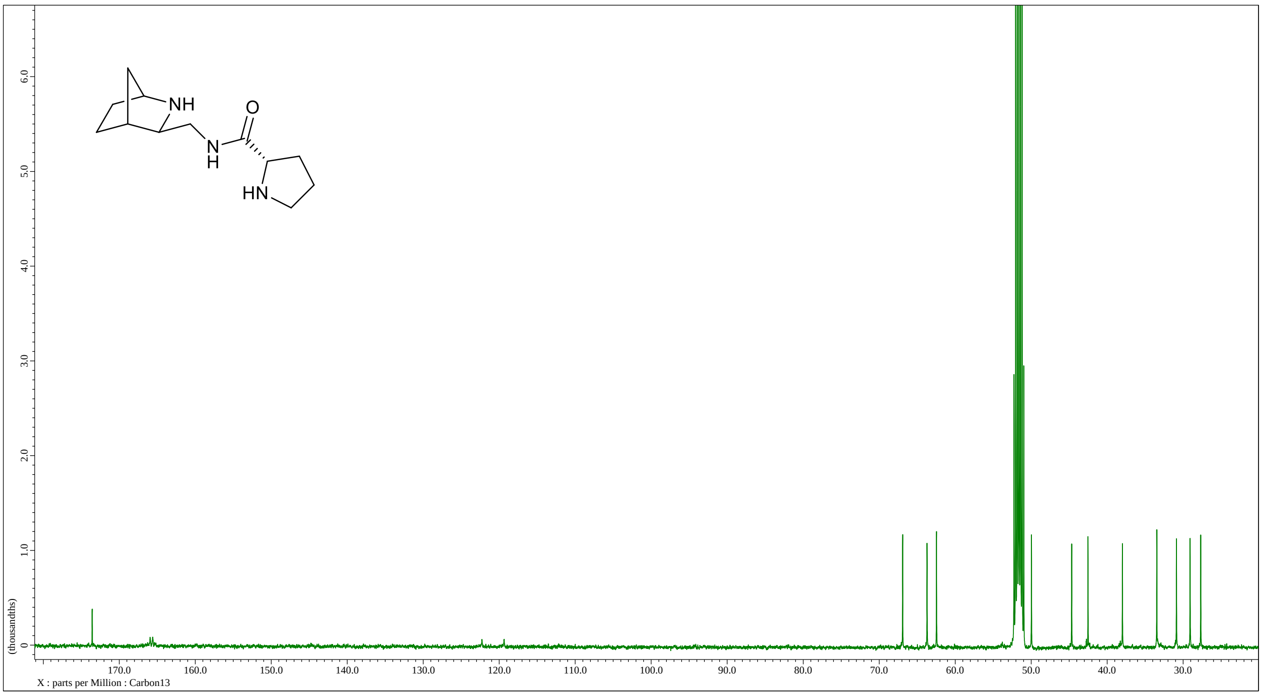


$^{13}$C NMR spectrum of *N*-((1*S*,3*R*,4*R*)-2-azabicyclo[2.2.1]heptan-3-ylmethyl)pyrrolidine-2-carboxamide (**8**)

Colorless crystals . Yield 84.5 mg (64%), mp. 65℃, $[\alpha]_D^{20}$ = -26.3 (c 0.0057, MeOH). $^1$H NMR (400 MHz, MeOH-$d_4$): δ 1.53-1.57 (m, 1H), 1.71-1.94 (m, 6H), 2.02-2.11 (m, 3H), 2.41-2.48 (m, 1H), 2.58 (br s, 1H), 3.34-3.50 (m, 4H), 4.10 (br s, 1H), 4.30-4.34 (m, 1H) ppm. $^{13}$C NMR (400 MHz, MeOH-$d_4$): δ 27.7, 29.1, 30.9, 33.5, 38.0, 42.5, 44.7, 50.0, 62.4, 63.7, 66.9, 173.6 ppm. HRMS (ESI$^+$, m/z): calcd for $C_{12}H_{21}N_3O$ ([M+H]$^+$) 224.1763; found 224.1758.

*N-(((1S,3R,4R)-2-(1-phenylethyl)-2-azabicyclo[2.2.1]heptan-3-yl)methyl)pyrrolidine-2-carboxamide (**12**)*

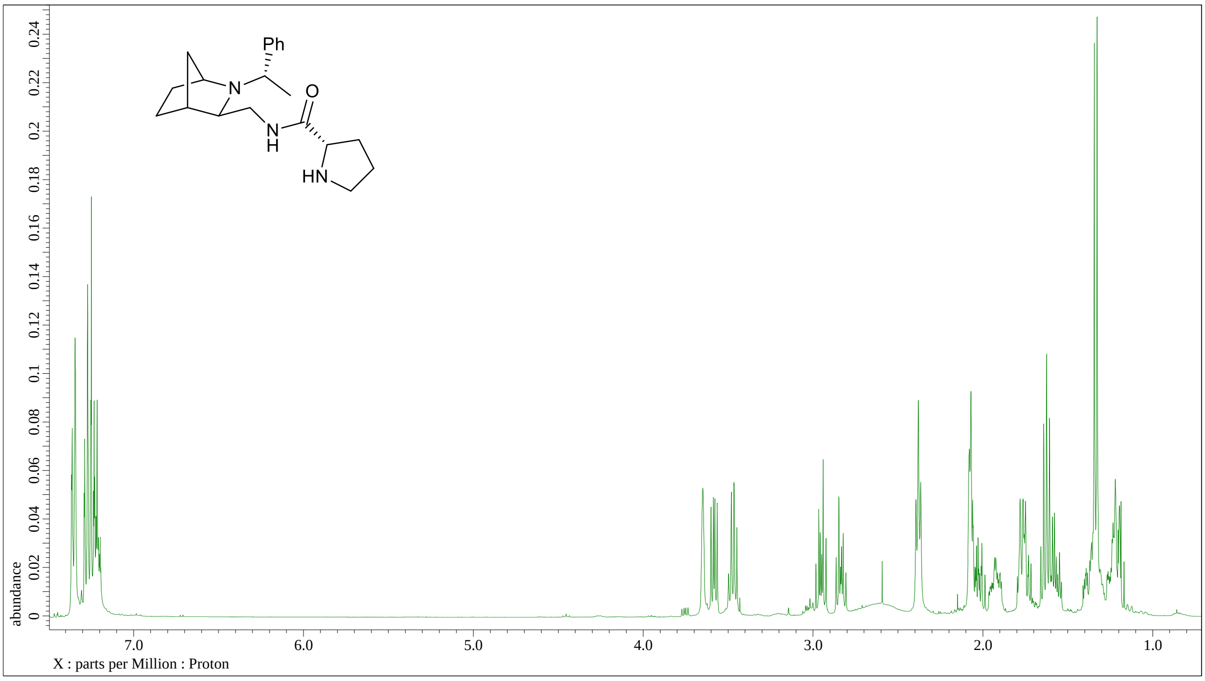


$^1$H NMR spectrum of *N*-(((1*S*,3*R*,4*R*)-2-(1-phenylethyl)-2-azabicyclo[2.2.1]heptan-3-yl)methyl)pyrrolidine-2-carboxamide (**12**)

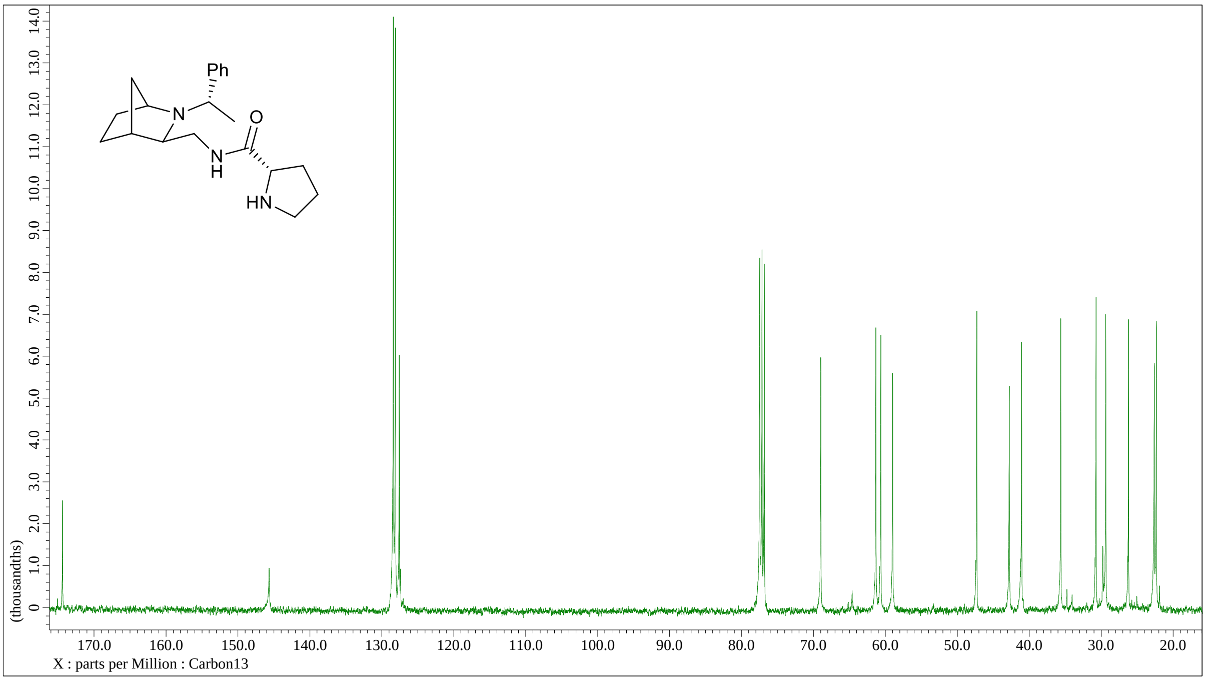

$^{13}C$ NMR spectrum of *N*-(((1*S*,3*R*,4*R*)-2-(1-phenylethyl)-2-azabicyclo[2.2.1]heptan-3-yl)methyl)pyrrolidine-2-carboxamide (**12**)

Yellow oil. Yield 0.39 g (62%). $[\alpha]_D^{20}$ = 42.6 (c 0.60, $CH_2Cl_2$). $^1H$ NMR (400 MHz, $CDCl_3$) δ =1.19-1.26 (m, 3H), 1.29-1.41 (m, 4H), 1.54-1.66 (m, 3H), 1.71-1.78 (m, 1H), 1.99-2.08 (m, 3H), 2.38 (t, 2H, *J* = 5,6 Hz), 2.59 (br s, 1H), 2.81-2.86 (m, 1H), 2.92-2.98 (m, 1H), 3.47 (q, 1H, *J* = 6.4 Hz), 3.56-3.60 (m, 1H), 3.65 (br s, 1H), 7.19-7.36 (m, 5H) ppm. $^{13}C$ NMR (75 MHz, $CDCl_3$) δ = 22.3, 22.6, 26.3, 29.4, 30.7, 35.6, 41.1, 42.8, 47.3, 59.0, 60.6, 61.3, 68.9, 127.6, 128.1, 128.4, 145.7, 174.4 ppm. HRMS (ESI$^+$): calcd for $C_{20}H_{29}N_3O$ $[M+H]^+$ 328.2389; found 328.2379.
